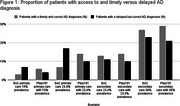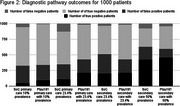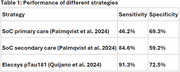# Evaluating the Impact on Diagnostic Performance and Healthcare Resource Utilization of Introducing a plasma rule‐out test in the Alzheimer's Disease Diagnostic Pathway

**DOI:** 10.1002/alz70856_102729

**Published:** 2025-12-24

**Authors:** Sophie Roth, Gustaf Ortsäter, Joana Amorim Freire

**Affiliations:** ^1^ Roche Diagnostics International AG, Rotkreuz, Zug, Switzerland; ^2^ Quantify Research AB, Stockholm, Stockholm, Sweden; ^3^ Roche Diagnostics International, AG, Rotkreuz, Zug, Switzerland

## Abstract

**Background:**

Availability of disease‐modifying therapies (DMTs) for Alzheimer's disease (AD) increases diagnostic demand. Prior to confirmatory testing with amyloid positron emission tomography or cerebrospinal fluid testing, amyloid pathology is not assessed in the AD diagnostic pathway. The current standard of care (SoC) includes clinical, cognitive and imaging tests. This study evaluates the impact on diagnostic performance and resource utilization of introducing a plasma rule‐out test for amyloid pathology, within the US healthcare system.

**Method:**

A cohort‐based model simulated 1000 patients being initially assessed for symptoms of subjective cognitive decline, mild cognitive impairment and mild AD in primary or secondary care, with the introduction of a plasma rule‐out test (Elecsys pTau181) compared to SoC prior to confirmatory testing. The following scenarios were evaluated based on the prevalence of amyloid pathology and setting for SoC and Elecsys ptau181: primary care with 10% prevalence, primary care with 23.4% prevalence, secondary care with 23.4% prevalence and secondary care with 50% prevalence. Prevalence and performance estimates were based on published literature (Quijano et al. 2024, Palmqvist et al. 2024). Outcomes measured included true and false positive/negative patients, healthcare resource utilization and the proportion of accurate and timely versus delayed AD diagnoses.

**Result:**

The plasma rule‐out test improved diagnostic outcomes across all scenarios. True positives increased by 98.5% while false negatives were reduced by 83.9% in primary care with 10% and 23.4% prevalence. False positives decreased by 32.5% and true negatives increased by 22.4% in secondary care with 23.4% and 50% prevalence. Across all scenarios, healthcare visits decreased by 1.8‐5.3%, and procedures decreased by 1.5‐3.6%. The highest increase in timely and correct AD diagnosis was 7% in primary care with 23.4% prevalence. Delayed accurate diagnoses was reduced by 7% in primary care with 10% prevalence. Overall, introducing a plasma rule out‐test in primary care scenarios showed better diagnostic outcomes, except for false positives and true negatives.

**Conclusion:**

Introducing a plasma rule‐out test in the AD diagnostic pathway improves diagnostic accuracy and reduces resource use, especially in low‐prevalence settings, aiding decision‐makers in refining clinical strategies.